# Adverse perinatal outcomes after Roux-en-Y Gastric Bypass vs. Sleeve Gastrectomy: a systematic review

**DOI:** 10.1186/s12884-023-05515-7

**Published:** 2023-08-02

**Authors:** Astrid Kistner, Alva Werner, Mehreen Zaigham

**Affiliations:** 1 Department of Obstetrics & Gynecology, Lund University and Skåne University Hospital, Malmö, 205 01 Sweden; 2 Obstetrics & Gynecology, Institution of Clinical Sciences Lund, Lund University, Lund, Sweden; 3 Program in Global Surgery and Social Change, Harvard Medical School, Boston, MA USA

**Keywords:** Roux-en-Y gastric bypass, Sleeve gastrectomy, Perinatal outcomes, Bariatric surgery, Mode of birth, Gestational age, Small for gestational age, Large for gestational age, Intrauterine death.

## Abstract

**Background:**

Pregnancies occurring after bariatric surgery are associated with various perinatal complications. However, there may be differences in the type of perinatal complications occurring after different methods of bariatric surgery. The aim of the current study was to compare adverse perinatal outcomes in pregnant women following Roux-en-Y Gastric Bypass (RYGB) vs. Sleeve Gastrectomy (SG).

**Methods:**

A systematic database search was performed in PubMed, Embase, Scopus and CINAHL. Observational studies comparing perinatal outcomes post-bariatric (RYGB and SG) surgery to pregnancies without prior surgery were selected. Outcomes of interest were: maternal body mass index (BMI) at the time of conception, mode of delivery, time from surgery to conception, birth weight, gestational age and intrauterine fetal death. Article selection, risk of bias assessment and data extraction, were performed by two authors. The study protocol was published in its revised form in PROSPERO, registration number: CRD42021234480.

**Results:**

A total of 3201 records were extracted. After duplicates were removed, 3143 records were screened for inclusion. Six studies fitted the selection criteria, of which four studies were RYGB and two SG (1100 post-RYGB vs. 209 post-SG). For the included studies, higher incidence of both SGA (22.9%, 11.9%, 14.2%) and LGA (4.2%, 4.8%, 1.7%) in SG compared to Roux-en-Y (SGA: 8.8%, 7.7%, 11.5%, 8.3% and LGA: 3.4%, 0.7%) were observed. SG had a shorter surgery to conception interval as compared to RYGB. Risk of bias assessment was moderate to serious for the studies included in the review, with bias in selection of participants being the major reason.

**Conclusion:**

Our systematic review demonstrated no major differences in BMI, mode of delivery, birthweight, gestational age, or rates of intrauterine death between women having undergone RYGB vs. SG. The rate of SGA and LGA births were higher in the SG group, but this group also had a shorter surgery to conception interval. Future studies are indicated to counsel women of reproductive age on the most appropriate type of bariatric surgery that is associated with the best perinatal outcomes.

**Supplementary Information:**

The online version contains supplementary material available at 10.1186/s12884-023-05515-7.

## Background

Obesity is a growing global health pandemic and it is estimated that approximately 39% of adults over the age of 18 suffer from the condition [1]. Among obese women, infertility is a common issue and studies show that women are strongly over-represented among patients undergoing bariatric surgery [2]. Significant weight loss following bariatric surgery often results in these women regaining fertility shortly after surgery [3]. Whilst maternal health may be improved, there is a large body of evidence that has found an increased risk of perinatal complications secondary to bariatric surgery like preterm birth, small for gestational age (SGA), large for gestational age (LGA), perinatal mortality among other adverse outcomes [3, 4]. However, results may vary markedly between studies and the type of bariatric surgery performed [3, 4]. While gastric bypass, also known as Roux-en-Y Gastric Bypass (RYGB), is a well-studied procedure, there are other procedures that are becoming more popular including Sleeve Gastrectomy (SG) and gastric banding (GB) [5].

RYGB is a restrictive-malabsorptive bariatric surgery that, up until recently, has been the most common bariatric procedure [6]. A small gastric pouch is created at the top of the ventricle and the lower end of the small intestine is connected to the gastric pouch. The small size of the pouch restricts food intake substantially, while the bypass past the small intestine, hinders absorption. RYGB can result in 60–70% loss of excess body weight [7]. SG is a restrictive procedure where most of the ventricle is removed, and much like RYGB, helps the patient to feel full with less food. SG is becoming increasingly popular as it has been associated with less adverse outcomes yet yields similar weight loss results as compared to RYGB [8]. However, there is a lack of long-term outcomes recorded in SG as compared to RYGB since RYGB has been performed over a longer period of time and in a larger group of patients [9].

As such, comparisons of adverse perinatal outcomes between RYGB and SG are important to enable women to make informed choices regarding type of surgery since fertility is an important reason for undergoing bariatric surgery in the first place. To the best of our knowledge, there are currently no systematic comparisons between the two techniques with respect to adverse perinatal outcomes. Therefore, the objective of this systematic review was to compare adverse perinatal outcomes following RYGB vs. SG in pregnant women. More specifically, we wanted to establish whether mode of delivery and maternal body mass index (BMI) differed in women after RYGB vs. SG surgery, and the incidence of adverse perinatal outcomes including low birthweight (in grams), gestational age (in days) and intrauterine fetal death after RYGB vs. SG surgery.

## Methods

### Data sources

The study protocol was published in its revised form in PROSPERO, registration number: CRD42021234480 [10]. An extensive systematic literature search was conducted using PubMed, Embase, Scopus and CINAHL by an experienced librarian (K.A) at Lund University during April 27, 2021 to May 5, 2021 (Search strategy, Table S1). This search did not include Cochrane Central. It was later updated to include records up to January 2, 2023 (Search strategy, Table S2) according to Bramer et al. [11]. All records identified by the search strategy were uploaded to Covidence, a software for managing systematic reviews [12]. Two authors (A.W and A.K), individually screened all the records based on inclusion and exclusion criteria as outlined by the study protocol. The selection of articles was done by screening for title and abstract and later on by reading full texts. A Preferred Reporting Items for Systematic Reviews (PRISMA) flow chart [13] was constructed to depict the flow of information through the different phases of the systematic review. In case of disagreement based on eligibility or uncertainty regarding an article, a third more experienced researcher (M.Z) was asked for a decision. Articles were therefore screened and selected by A.W and A.K based on relevance and quality. There were four articles that fitted the selection criteria based on their title and abstract but full-texts were not available in the published literature (Table S3). Extensive efforts were made to extract the full-texts including contacting Lund University library services, email contact with journals in which the articles were published and even attempts to reach the study authors by email and social media, but unfortunately without any success.

### Eligibility criteria

The selection criteria were predefined before the initial screening process. Since bariatric surgery is a relatively new surgical method, there were no restrictions with respect to publication date. Similarly, no restrictions regarding country, language or study type were implemented. Observational studies were included, such as: cross sectional, case control and cohort studies. Studies comparing perinatal outcomes, post-bariatric (SG and RYGB) surgery to pregnancies without prior bariatric surgery (SG and RYGB) were selected. Inclusion criteria required a control group (pregnancies with no history of bariatric surgery) to be present in the studies finally selected for the review. Time from operation to conception was limited to three years since we wanted to study perinatal outcomes in the few years after bariatric surgery. Maternal and perinatal outcomes of interest were: maternal BMI at the time of conception, mode of delivery, time from surgery to conception, birth weight, gestational age and intrauterine fetal death. Selected studies had to report all these outcomes to be included in the final review.

After screening all articles based on the inclusion and exclusion criteria, it was discovered that the selection criteria were too strict for studies pertaining to SG surgery. SG had only recently become more common than RYGB [6] and large studies outlining perinatal outcomes after SG were therefore limited. The study group therefore decided to deviate from the criteria: “time from operation to conception to a maximum of 3 years” to a mean interval below 4 years for all studies, both SG and RYGB. Another exception was made regarding the requirement that studies had to include all outcomes of interest, since there was only one study [14] about SG which did report birthweight in the results. However, the study [15] that did not mention birth weight, did include small for gestational age (SGA) and large for gestational age (LGA) which are outcomes calculated based on birthweight and gestational age. Therefore, SGA and LGA were added to the study outcomes. This exception was made because of the low number of studies on SG that fitted into our selection criteria.

### Risk of bias assessment

The “Risk of Bias in Non-randomized Studies - of Interventions” (ROBINS-I) tool was used for the risk of bias assessment [16]. A.W and A.K performed the risk of bias assessment independently and assessed the following domains: bias due to confounding, bias in selection of participants into the study, bias in classification of interventions, bias due to deviations from intended interventions, bias due to missing data, bias in measurement of outcomes, bias in selection of the reported result and overall risk of bias. Confounding factors were stated beforehand: time after bariatric surgery, parity, smoking, BMI, maternal age, gestational diabetes mellitus (GDM) and hypertension. The risk of bias assessment was conducted before extracting the data to minimize the risk of authors bias due to the results reported in the studies. After performing the risk of bias assessment individually, A.W and A.K discussed all articles and agreed upon a final decision regarding each domain. If there was disagreement, a third review (M.Z) was consulted.

### Data collection and analysis

Data was extracted individually by two authors (A.W and A.K) using a specifically developed data extraction form in Microsoft Excel [17]. Data extracted consisted of: authors, publication date, country of study, study design, quality of study, population characteristics, maternal BMI, mode of delivery, time interval from surgery to conception, inclusion and exclusion criteria, known confounders, adverse fetal outcomes and exposure (RYGB vs. SG and obesity in the comparison group of women without bariatric surgery). Outcomes of perinatal morbidity and mortality were: gestational age, birth weight, SGA, LGA and intrauterine fetal death. Mean values, standard deviations (SD), median values, CI and Odds Ratios (OR) were extracted if available.

## Results

**Fig. 1:**
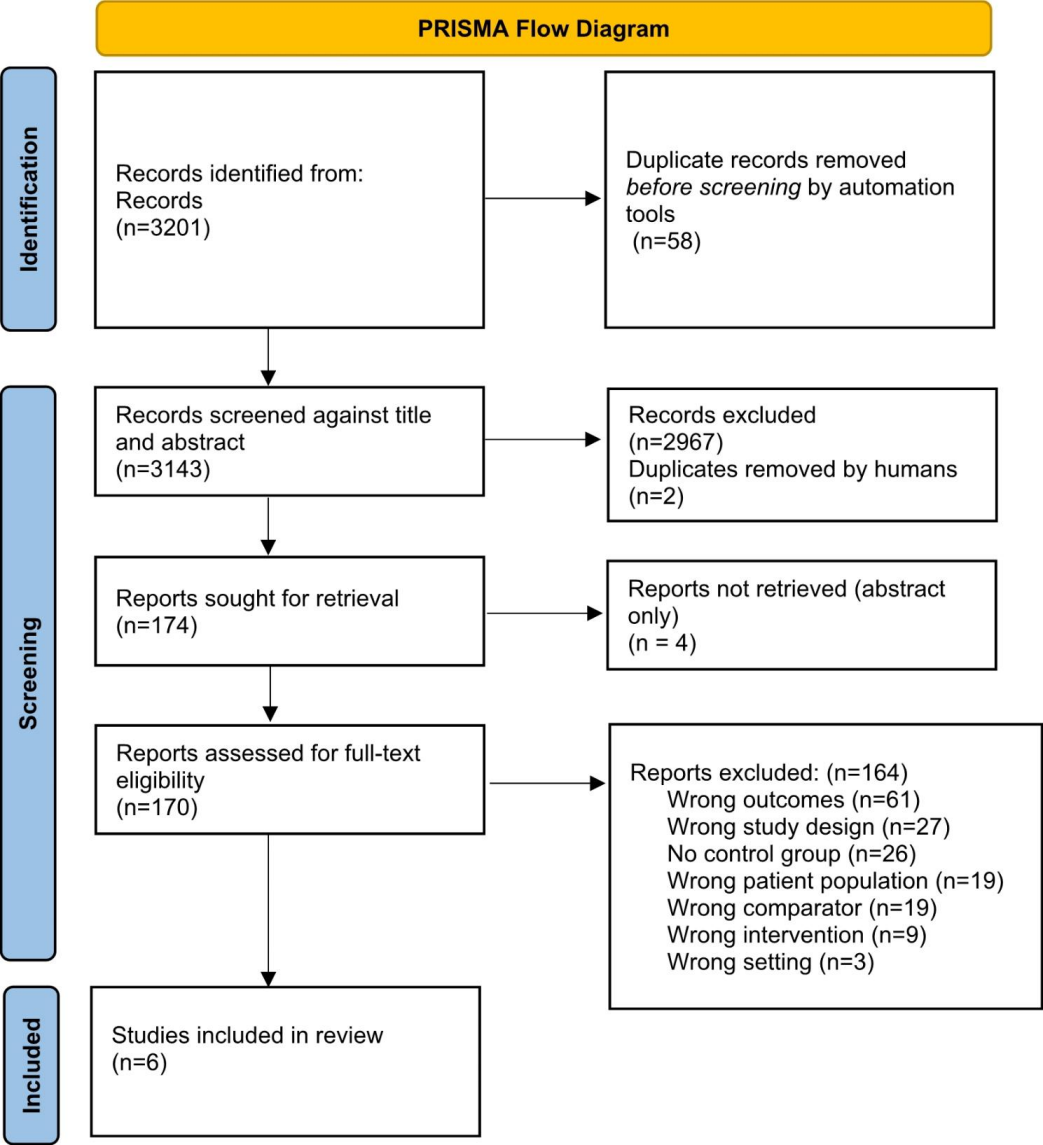
PRISMA Flow Chart [13] Flow of information in the review showing the number of records identified, included and excluded, and the reasons for exclusions

The search generated 3201 records and after duplicates were removed, there were 3143 records to screen against title and abstract. A total of 2969 records were excluded because of irrelevancy and thereafter 174 reports were sought for retrieval of which 170 went on to full-text screening. Reasons for exclusion are shown in Fig. 1. Characteristics of the studies included in the review are presented in Table 1 with 1100 post-RYGB and 209 post-SG participants. The overall risk of bias assessment, Table 2, for most articles was moderate or serious. The major problems of the included studies were bias due to confounding and bias in selection of participants.

**Table 1:** Characteristics of the Roux-en-Y Gastric Bypass and Sleeve Gastrectomy studies included in the review

Type of Bariatric Surgery	Author	Name of study	Country	Year of enrollment	Study design	Sample size	Control sample size
Roux-en-Y Gastric Bypass	Adams et al. [18]	Maternal and neonatal outcomes for pregnancies before and after gastric bypass surgery	USA	2015	Retrospective cohort	764	764
	Kjær et al. [19]	The risk of adverse pregnancy outcome after bariatric surgery: a nationwide register-based matched cohort study	Denmark	2013	Matched cohort study	286	1070
	Patel et al. [21]	Pregnancy outcomes after laparoscopic Roux-en-Y gastric bypass	USA	2008	Retrospective cohort	26	254
	Santulli et al. [20]	Obstetrical and neonatal outcomes of pregnancies following gastric bypass surgery: a retrospective cohort study in a French referral centre	France	2010	Retrospective cohort	24	120
Sleeve Gastrectomy	Karadağ et al. [15]	Effects of laparoscopic sleeve gastrectomy on obstetric outcomes within 12 months after surgery	Turkey	2019	Retrospective observational study	90	54
	Rottenstreich et al. [14]	Maternal and Perinatal Outcomes After Laparoscopic Sleeve Gastrectomy	USA	2018	Retrospective case control	119	119

**Table 2:** Risk of Bias assessment with ROBINS- I

Article	Bias due to confounding	Bias in selection of participants	Bias in classification of interventions	Bias due to deviations from intended interventions	Bias due to missing data	Bias in measurement of outcomes	Bias in selection of the reported result	Overall risk of bias
Adams et al. [18]	Moderate (a)	Moderate	Low	Low	Moderate (b)	Low	Low	Moderate
Karadağ et al. [15]	Serious (c)	Moderate (d)	Low	Low	Moderate	Low	Low	Serious
Kjær et al. [19]	Moderate	Critical (e)	Low	Low	Low	Moderate	Serious	Critical
Patel et al. [21]	Serious (f)	Moderate	Low	Low	Low	Low	Low	Serious
Rottenstreich et al. [14]	Serious (g)	Moderate	Low	Low	Low	Low	Low	Serious
Santulli et al. [20]	Moderate	Moderate	Low	Low	Low	Moderate (h)	Low	Moderate

(a) Matched control group. Logistic regression adjusted for several known confounders

(b) Mode of delivery only reported for half of the sample size of interest

(c) No adjustment for maternal age

(d) Excluded multiple pregnancies, miscarriages, and intrauterine fetal demise

(e) Merged RYGB and GB as bariatric surgery outcomes in some of their results

(f) No adjustment for maternal age

(g) No adjustment for smoking

(h) Some missing data were collected by telephone interviews

In Table 3, maternal characteristics are presented. Adams et al. [18] and Kjaer et al. [19] were the only studies that presented OR. Among maternal outcomes, there were generally small differences between case and control groups, suggesting that maternal BMI, vaginal birth rates and number of Cesarean sections were homogenous between the groups. Time from surgery to conception varied greatly between the studies and was in general shorter in women that underwent SG surgery as compared to RYGB (Table 3).

**Table 3:** Maternal characteristics based on type of bariatric surgery in the studies included in the review. All results are presented as mean value ± SD, n (%) and [CI] if nothing else is stated

Type of Bariatric Surgery	Article	Sample size (n)	Maternal BMI at the time of conception	Vaginal birth	Instrumental vaginal birth	Cesarean section	Time from surgery to conception (months)
Roux-en-Y Gastric Bypass	Adams et al. [18]	295	28.6 ± 5.7	177 (60%)	12 (4.1%)	106 (35.9%)	50.4 ± 38.4 ^**a**^
	Kjær et al. [19]	286	32.4	228 (79.7%)	NI	111 (32.7%)	Median 20.6 [3-113] ^**a**^
	Patel et al. [21]	26	32.5 ± 7.2	10 (38.5%)	NI	16 (61.5%)	25.4 ± 13
	Santulli et al. [20]	24	32.7 +- 5.4	14 (58.3%)	2 (8.3%)	8 (33.3%)	26.6 [3–74]
Sleeve Gastrectomy	Karadağ et al. [15] group A ^**b**^	48	(A) 32.83 ± 3.63	24 (50%)	NI	24 (50%)	7.8 ± 3.4
	Karadağ et al. [15] group B ^**b**^	42	(B) 28.9 ± 2.84	22 (52.4%)	NI	20 (47.6%)	25.8 ± 3.4
	Rottenstreich et al. [14]	119	29.5 [26.6–32.0]	73 (61.3%)	4 (3.4%)	42 (35.3%)	16.7 [12–31]

NI = No information

^**a**^ Values reported as time from surgery to delivery

^**b**^ In Karadağ et al. [15], patients were divided into group A and group B based on the time interval from surgery to conception, therefore results are reported separately

In the study by Kjaer et al. [19], no CI or SD was reported for maternal BMI, only mean values were given. Furthermore, some results were reported as a “Bariatric surgery group”, meaning a population of both RYGB and Gastric Banding (GB) patients. Mode of delivery, time from surgery to conception and intrauterine death were the parameters reported in this way. There were 339 patients, 84% of which were RYGB patients and 16% were GB patients. Kjaer. [19] reported time from surgery to delivery instead of surgery to conception. The results were presented in days instead of months, and were thus converted to median time from surgery to delivery in months instead of days. In the study by Adams et al. [18], the parameter was “surgery to birth (4.2 ± 3.2 years)” instead of surgery to conception and results were presented as years, which were converted into months. Adam et al. [18] looked at different patient groups: group 1, 2 and 3. For this study, we extracted details with regards to group 2, since this was the only group that fitted the criteria. However, mode of delivery was not documented for all women in Group 2 and therefore only n = 295 were included in this systematic review (and not the entire sample size of n = 764).

Cesarean section was either recorded separately as before or during labor [14] or as Yes/No [15, 17–20]. There was therefore no information on whether these Cesareans were elective or medically motivated.

Perinatal outcomes including birth weight, SGA, LGA, gestational age and intrauterine death are shown in Table 4. Adams et al. [18], Kjaer et al. [19], Santulli et al. [20] and Rottenstreich et al. [14] showed significantly lower birth weight in the bariatric surgery operated women compared to control groups (*P* < 0.0001, *P* < 0.001, *P* < 0.0001, *P* = 0.001). Patel et al. [19] did not have significant results for the difference in birth weight between the groups (*P* = 0.361).

**Table 4:** Perinatal outcomes based on type of bariatric surgery in the studies included in the review. All results are presented as mean value ± SD, n (%) and [CI] if nothing else is stated

Surgery	Article	Sample size (n)	Birth weight (grams)	LGA	SGA	Gestational age (weeks)	Intrauterine death
Roux-en-Y gastric bypass	Adams et al. [18]	764	3093 ± 568	10 (3.4%)	26 (8.8%)	38.38 ± 2.34	1
	Kjær et al. [19]	286	3264 ± 566	2 (0.7%)	22 (7.7%)	39.14 ± 1.97	1
	Patel et al. [21]	26	2951 ± 646	NI	3 (11.5%)	37 ± 2.6	0
	Santulli et al. [20]	24	2948.2 ± 435	NI	2 (8.3%)	39.1 ± 1.4	2 pregnancies excluded due to miscarriages
Sleeve Gastrectomy	Karadağ et al. [15] group A	48	NI	2 (4.2%)	11 (22.9%)	37.9 ± 2.5	Excluded fetal death ^**a**^
	Karadağ et al. [15] group B	42	NI	2 (4.8%)	5 (11.9%)	38.3 ± 2.4	Excluded fetal death ^**a**^
	Rottenstreich et al. [14]	119	3002 [2765–3262] ^**b**^	Median 2 (1.7%)	Median 17 (14.2%)	38.9 [38–39.9] ^**b**^	1

NI = No information

^**a**^ Pregnancies with intrauterine death were excluded from the study

^**b**^ Interquartile range

In Adams et al. [18], the RYGB group (group 2) had a significantly lower incidence of LGA (*P* < 0.0001, OR = 0.33 [0.21–0.51]) and higher incidence of SGA (*P* = 0.0003, OR = 2.16 [1.43–3.32]) compared to controls. In Kjaer et al. [19], the RYGB group had a significantly lower frequency of LGA (*P* = 0.001, OR = 0.09 [0.02–0.36]) and higher frequency of SGA (*P* = 0.001, OR = 2.78 [1.56–4.96]) compared to controls. Patel et al. [21] and Santulli et al. [20] showed no significant results in incidences of SGA between the RYGB group and the control group. In group A of Karadag et al. [15], the SG group had a significantly lower incidence of LGA (*P* < 0.05) and a higher incidence of SGA (*P* < 0.05). In group B of Karadag et al. [15], the SG group had a significantly lower incidence of LGA (*P* < 0.05), although not significantly higher incidence of SGA compared to the control group. The SG group in Rottenstreich et al. [14] had significantly lower rates of LGA (*P* = 0.001) and higher rates of SGA (*P* = 0.01) compared to controls.

For Kjaer et al. [19], the mean gestational age was significantly lower compared to the control group (*P* < 0.001). In the other studies, no significant results according to gestational age were observed between the groups. There were no significant results in any of the studies for intrauterine death.

## Discussion

This systematic review aimed to compare perinatal outcomes after two major types of bariatric surgery, namely RYGB and SG. Although our review did not demonstrate any differences in BMI or mode of delivery between RYGB operated women and SG operated women, our results showed similar results of birthweight and gestational age, as well as rates of intrauterine death between the groups. We noticed higher rates of both SGA (22.9%, 11.9%, 14.2%) and LGA (4.2%, 4.8%, 1.7%) in SG compared to Roux-en-Y group (SGA: 8.8%, 7.7%, 11.5%, 8.3% and LGA: 3.4%, 0.7%). However, there was also a tendency toward shorter surgery to conception intervals in these studies, which might be associated with higher rates of perinatal complications. As shown in group A in Karadag et al. [15], the shortest surgery to conception interval (7.8 ± 3.4 months) was for SG which was also associated with the highest rate of SGA infants (22.9%). The adverse impact of a shorter interval was strengthened by a systematic review by Akhter et al. [4] who concluded that SGA occurs more frequently in malabsorptive procedures as compared to restrictive procedures, thus suggesting conflicting results from our study. However, the higher rate of LGA in the SG group did correlate with Akhter et al. [4] and important maternal characteristics, such as advanced maternal age, BMI > 30, alcohol, and smoking may act as confounders to adverse outcomes in the post-surgical group. Similarly, in our study the post-surgical groups often had a higher mean age than the control non-surgical groups [4, 17, 20].

To maximize the quality of the study, the Cochrane standardized protocol for systematic reviews was followed. The study protocol was published in PROSPERO before the start of the review [10] and a Risk of Bias assessment via the ROBINS-I tool [16] was performed which helped minimize the influence of bias in our study. A thorough literature search was also performed so as to make sure no relevant articles would be missed. In our Risk of Bias assessment, most articles were assessed to have a moderate or serious overall risk of bias. This did not mean that these articles were of low quality, but rather that risk of biases are always a factor to consider with cohort and case-control studies. Due to the nature of our research questions, randomized control trials (RCTs) were not available since no studies were found that compared the interventions solely to compare perinatal outcomes. A meta-analysis was not conducted mainly due to the handful of studies that fit the search criteria and heterogeneity in the comparisons being made by the primary studies. Critical differences, for example, were found for the ‘time from surgery to conception’, which varied greatly between the studies and was in general shorter in women that underwent SG surgery as compared to RYGB. The risk of bias assessment was also considered in this regard, as we anticipated that it would simply compound possible errors and produce results that may be interpreted incorrectly.

BMI at conception were similar in all study groups but due to the confounding effect of surgery to conception interval, we reported separate results for different groups from Karadag et al. [15], as we wanted to highlight the influence of this interval. Another weakness was the differences in total populations in the two intervention groups compared. As Cochrane protocol were strictly followed, the exclusion criteria were decided before performing the database search. This unfortunately resulted in a relatively small selection of SG studies leading to a smaller population of post-SG mothers included. This limited the possibilities to draw conclusions based on the studies included in the review. Similarly, there were differences in time interval from surgery to conception between the different studies. Conception during the malnutrition period after bariatric surgery is known to affect perinatal outcomes and therefore it is not representable to compare groups with greater differences in interval [21].

As mentioned, deviations from the study protocol were also made and an early detection of the very small sample with due to strict surgery to conception interval led to a collective decision to change this exclusion criteria from a maximum of 3 years to a mean smaller than 4 years by the study group. This was decided early on, and no articles were excluded by the 3-year interval criteria before the deviation from the protocol.

There were several aberrations in type of values reported in the different studies. Kjaer et al. [19] did not report a SD or CI for maternal BMI at conception and then used median instead of mean values. The study also reported some outcomes for RYGB and some for RYGB and Gastric banding, resulting in data that could not be added to the current review. Some articles did not report on all outcomes described in the introduction and method. For example, Karadag et al. [15] did not report birthweight even though they reported SGA and LGA which requires birthweight to be calculated.

Systematic reviews are meant to develop and improve patient care and can form the basis for clinical practice. Our goal with this review was to identify which type of bariatric was associated with the least adverse perinatal outcomes. This would have enabled clinicians to provide the best potential care for their patients and give the patients the opportunity to be involved in their course of treatment for the best possible perinatal outcome.

## Conclusion

In this systematic review, we demonstrated similar results between pre-pregnancy BMI, mode of delivery, birthweight, gestational age and rates of intrauterine death in both RYGB and SG operated women. Higher rates of SGA and LGA were found in the SG group, however, a possible major confounding factor was the time interval from surgery to conception which was found to be shorter in the SG group. This, together with other confounding factors such as maternal age, parity, smoking and alcohol; may strongly affect the risk of maternal-fetal and neonatal complications. Future studies are indicated to establish which type of bariatric surgery is associated with the least adverse perinatal outcomes.

### Electronic supplementary material

Below is the link to the electronic supplementary material.

**Additional file 1: Table S1.** Database search strategy for items up to 2021-04-28

**Additional file 2: Table S2.** Updated database search strategy for items from 2021-04-28 to 2023-01-02

**Additional file 3: Table S3.** References of reports with no accessible full text

## Data Availability

The datasets used and/or analyzed during the current study are available from the corresponding author on reasonable request.

## Abbreviations

BMI: Body mass index
CI: Confidence interval
GB: Gastric Banding
GDM: Gestational Diabetes Mellitus
LGA: Large for gestational age
OR: Odds ratio
PRISMA: Preferred Reporting Items for Systematic Reviews
RCT: Randomized controlled trial
ROBINS-I: Risk of Bias in Non-randomized Studies- of Interventions
RYGB: Roux-en-Y Gastric Bypass
SD: Standard deviation
SG: Sleeve Gastrectomy
SGA: Small for gestational age
