# Supplementary material for: Adverse perinatal outcomes after Roux-en-Y Gastric Bypass vs. Sleeve Gastrectomy: a systematic review
Source: BMC Pregnancy Childbirth. 2023 Aug 2;23:557. doi: 10.1186/s12884-023-05515-7 (PMC10394842; doi:10.1186/s12884-023-05515-7)
Supplement: Supplementary file 1 — Additional file 1: Table S1. Database search strategy for items up to 2021-04-28 [file 12884_2023_5515_MOESM1_ESM.docx]

**Table S1.**

Database search strategy for items up to 2021-04-28

| Krister Aronsson, Medicinska fakulteten  Krister.aronsson@med.lu.se | 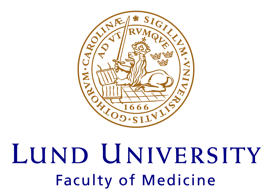 |
| --- | --- |

**Sökrapport**

# **Projektinformation**

| beställningsdatum | beställarens namn | institution/organisation |
| --- | --- | --- |
| 2021-04-27 | Mehreen Zaigham | Medicinska fakulteten |
|  |  |  |

# Tidsplan för projektet - Önskat datum för slutleverans 2021-05-05

# BESKRIV FRÅGESTÄLLNINGEN OCH SYFTET MED SÖKNINGEN

Comparison of Gastric bypass or gastric sleeve operation on pregnant women. A look mainly at adverse effects.

# Söktermer (PÅ engelska)

| gastric sleeve, gastric bypass, sleeve gastrectomy, biliopacreatic diversion, restrictive surgery, malabsorptive surgery, pregnancy, neonatal intensive care, gestational age, congential abnormalities, hypoxic-ischemic encephalopathy, birthweight |  |  |  |  |
| --- | --- | --- | --- | --- |
| **P**atient / population / problem |  |  | **P**opulation |  |
| **I**ntervention |  |  | **E**xposure |  |
| **C**omparison / control |  |  | **O**utcome |  |
| **O**utcome |  |  |  |  |
| **S**tudietyp |  |  |  |  |
|  |  |  |  |  |

# Referenser till nyckelartiklar – som motsvarar sådana artiklar som du vill att sökningen ska inkludera (3-5 artiklar)

Akhter Z, Rankin J, Ceulemans D, Ngongalah L, Ackroyd R, Devlieger R, Vieira R, Heslehurst N. Pregnancy after bariatric surgery and adverse perinatal outcomes: A systematic review and meta-analysis. PLoS Med. 2019 Aug 6;16(8):e1002866. doi: 10.1371/journal.pmed.1002866. PMID: 31386658; PMCID: PMC6684044.

Roos N, Neovius M, Cnattingius S, Trolle Lagerros Y, Sääf M, Granath F, Stephansson O. Perinatal outcomes after bariatric surgery: nationwide population based matched cohort study. BMJ. 2013 Nov 12;347:f6460. doi: 10.1136/bmj.f6460. PMID: 24222480; PMCID: PMC3898199.

Rottenstreich A, Elchalal U, Kleinstern G, Beglaibter N, Khalaileh A, Elazary R. Maternal and Perinatal Outcomes After Laparoscopic Sleeve Gastrectomy. Obstet Gynecol. 2018 Mar;131(3):451-456. doi: 10.1097/AOG.0000000000002481. PMID: 29420411.

# Önskat format för leverans av resultat (Endnote, pdf, word):

Endnote

Sökscheman

**2021-04-28**

**Pubmed**#1

("Gastric Bypass"[Mesh]) OR "Bariatric Surgery"[Mesh]
=28020

#2

bariatric surger*[Title/Abstract] OR gastric bypass*[Title/Abstract] OR gastric sleeve*[Title/Abstract] OR sleeve gastrectom*[Title/Abstract] OR laparoscopic gastrectom*[Title/Abstract] OR LSG[Title/Abstract] OR restrictive surger*[Title/Abstract] OR malabsorptive surger*[Title/Abstract] OR BPD[Title/Abstract] OR SG[Title/Abstract] OR biliopancreatic diversion*[Title/Abstract]
=49607

#3
#1 OR #2
=59146

#4
("Pregnancy"[Mesh]) OR "Pregnant Women"[Mesh]
=916300

#5
pregnanc*[Title/Abstract] OR pregnant[Title/Abstract]
=539741

#6
#4 OR #5
=1037006

#7
#3 AND #6
=2340

#8
((("Intensive Care, Neonatal"[Mesh]) OR "Gestational Age"[Mesh]) OR "Hypoxia-Ischemia, Brain"[Mesh]) OR "Congenital Abnormalities"[Mesh]
=689213

#9
time after operation[Title/Abstract] OR "weight loss" after operation[Title/Abstract] OR perinatal outcome*[Title/Abstract] OR perinatal complication*[Title/Abstract] OR perinatal morbidit*,[Title/Abstract] OR gestational age[Title/Abstract] OR gestational ages[Title/Abstract] OR birthweight[Title/Abstract] OR birth weight[Title/Abstract] OR congenital anomal*[Title/Abstract] OR SGA[Title/Abstract] OR LGA[Title/Abstract] OR hypoxic ischemic encephalopath*[Title/Abstract] OR HIE[Title/Abstract] OR seizures neonatal intensive care unit[Title/Abstract] OR NICU[Title/Abstract] OR GWG[Title/Abstract] OR gestational weight gain[Title/Abstract] OR WMD[Title/Abstract] OR weighted mean difference[Title/Abstract]
=184918

#10
#8 OR #9
820255

#11
#7 AND #10
=1210 referenser

#12
“case report”[Title/Abstract]
=335233

#13
#11 NOT #12
=1203

**2021-04-29**

**Embase**
#1

'gastric bypass surgery'/exp OR 'gastric sleeve'/exp OR 'bariatric surgery'/exp OR 'roux-en-y gastric bypass'/exp OR 'bariatric surger*':ab,ti OR 'gastric bypass*':ab,ti OR 'gastric sleeve*':ab,ti OR 'sleeve gastrectom*':ab,ti OR 'laparoscopic gastrectom*':ab,ti OR lsg:ab,ti OR 'restrictive surger*':ab,ti OR 'malabsorptive surger*':ab,ti OR bpd:ab,ti OR sg:ab,ti OR 'biliopancreatic diversion':ab,ti
=91891

#2
'pregnancy'/exp OR 'pregnant woman'/exp OR pregnanc*:ab,ti OR pregnant:ab,ti
=1049726

#3
#1 AND #2
=3556

#4
'newborn intensive care'/exp OR 'congenital disorder'/exp OR 'hypoxic ischemic encephalopathy'/exp OR 'gestational age'/exp OR 'time after operation':ab,ti OR 'weight loss after operation':ab,ti OR 'perinatal outcome*':ab,ti OR 'perinatal complication*':ab,ti OR 'perinatal morbidit*':ab,ti OR 'gestational age':ab,ti OR 'gestational ages':ab,ti OR birthweight:ab,ti OR 'birth weight':ab,ti OR 'congenital anomal*':ab,ti OR sga:ab,ti OR lga:ab,ti OR 'hypoxic ischemic encephalopath*':ab,ti OR hie:ab,ti OR 'seizures neonatal intensive care unit':ab,ti OR nicu:ab,ti OR gwg:ab,ti OR 'gestational weight gain':ab,ti OR wmd:ab,ti OR 'weighted mean difference':ab,ti
=1809985

#5
#3 AND #4
=2030

#6
#5 AND [embase]/lim NOT ([embase]/lim AND [medline]/lim)
=990

#7
'case report':ab,ti
=460998

#8
#6 NOT #7
=970 referenser

**Scopus**
#1

TITLE-ABS-KEY ( "gastric bypass*" OR "gastric sleev*" OR "bariatric surger*" OR "sleeve gastrectom*" OR lsg OR "restrictive surger*" OR "malabsorptive surger" OR "biliopancreatic diversion*" OR lsg OR bpd OR sg OR gs )
=142897

#2
pregnanc* OR pregnant
=1148673

#3
#1 AND #2
=3379

#4
TITLE-ABS-KEY ( "neonatal intensive care" OR "newborn intensive care" )

#5
TITLE-ABS-KEY ( "congenital disorder*" OR "congenital anomali*" )

#6
( TITLE-ABS-KEY ( "hypoxic ischemic" OR hypoxic-ischemic ) ) AND ( TITLE-ABS-KEY ( encephalopath* ) )

#7
TITLE-ABS-KEY ( weightloss OR "weight loss" OR birthweight OR "birth weight" )

#8
TITLE-ABS-KEY ( “perinatal AND outcome*” OR “perinatal AND complication*” OR “perinatal AND morbidit*” )

#9
TITLE-ABS-KEY ( "gestational age" OR "gestational ages" )

#10
TITLE-ABS-KEY ( "gestational weight gain" OR gwg OR "weighted mean difference" OR wmd OR sga OR lga OR hie )

#11
TITLE-ABS-KEY ( "neonatal intensive care unit" OR nicu )

#12
#4 OR #5 OR #6 OR #7 OR #8 OR #9 OR #10 OR #11
=601668
#13
#3 AND #12
=1888

#14
TITLE-ABS-KEY ( "case report" )
=2599377

#15
#13 AND NOT #14
=1797 referenser

**CINAHL**
#1
MM bariatric surgery OR MM gastric bypass OR TI ( bariatric surger* OR gastric bypass* OR gastric sleev* OR GS OR sleeve gastrectom* OR LSG OR restrictive surgery OR malabsorptive surgery OR BDP OR SG OR biliopancreatic diversion* ) OR AB ( bariatric surger* OR gastric bypass* OR gastric sleev* OR GS OR sleeve gastrectom* OR LSG OR restrictive surgery OR malabsorptive surgery OR BDP OR SG OR biliopancreatic diversion* )
=13543

#2
MH (MH "Pregnancy+") OR TI ( pregnanc* OR pregnant ) OR AB ( pregnanc* OR pregnant )
=264218

#3
#1 AND #2
=483

#4
( (MM "Neonatal Intensive Care Nursing") OR (MM "Intensive Care Units, Neonatal") OR (MM "Hypoxia-Ischemia, Brain, Neonatal") OR (MM "Gestational Age") ) OR TI ( time after operation OR weight loss after operation OR perinatal outcome* OR perinatal complication* OR perinatal morbidit* OR gestational age OR gestational ages OR birthweight OR "birth weight" OR congenital anomal* OR congenital abnormalt* OR SGA OR LGA, OR hypoxic ischemic encephalopath* OR hypoxic-ischemic encephalopath* OR HIE OR seizures and neonatal intensive care unit OR NICU ORGWG OR gestational weight gain OR WMD OR weighted mean difference ) OR AB ( time after operation OR weight loss after operation OR perinatal outcome* OR perinatal complication* OR perinatal morbidit* OR gestational age OR gestational ages OR birthweight OR "birth weight" OR congenital anomal* OR congenital abnormalt* OR SGA OR LGA, OR hypoxic ischemic encephalopath* OR hypoxic-ischemic encephalopath* OR HIE OR seizures and neonatal intensive care unit OR NICU ORGWG OR gestational weight gain OR WMD OR weighted mean difference )
=80875

#5

#3 AND #4
=126 referenser

 **Antal referenser**

**Före dubblettkontroll: 4092
Efter dubblettkontroll: 2816 referenser**
