# Supplementary material for: Adverse perinatal outcomes after Roux-en-Y Gastric Bypass vs. Sleeve Gastrectomy: a systematic review
Source: BMC Pregnancy Childbirth. 2023 Aug 2;23:557. doi: 10.1186/s12884-023-05515-7 (PMC10394842; doi:10.1186/s12884-023-05515-7)
Supplement: Supplementary file 2 — Additional file 2: Table S2. Updated database search strategy for items from 2021-04-28 to 2023-01-02 [file 12884_2023_5515_MOESM2_ESM.docx]

**Table S2.**

Updated database search strategy for items from 2021-04-28 to 2023-01-02

Krister Aronsson

Librarian, Faculty of medicine, Lund university

[krister.aronsson@med.lu.se](mailto:krister.aronsson@med.lu.se)

sökrapport

# projektinformation

| beställningsdatum | beställarens namn | institution/organisation |
| --- | --- | --- |
| 2022-12-21 | Mehreen Zaigham | Medicinska fakulteten |
|  |  |  |

# tidsplan för projektet - önskat datum för slutleverans

## 2023-01-03

# BESKRIV FRÅGESTÄLLNINGEN OCH SYFTET MED SÖKNINGEN

Uppdateringssökning för perioden 20210428- av tidigare sökning för ” Comparison of Gastric bypass or gastric sleeve operation on pregnant women. A look mainly at adverse effects.”

# söktermer (PÅ engelska)

| GASTRIC SLEEVE, GASTRIC BYPASS, SLEEVE GASTRECTOMY, BILIOPACREATIC DIVERSION, RESTRICTIVE SURGERY, MALABSORPTIVE SURGERY, PREGNANCY, NEONATAL INTENSIVE CARE, GESTATIONAL AGE, CONGENTIAL ABNORMALITIES, HYPOXIC-ISCHEMIC ENCEPHALOPATHY, BIRTHWEIGHT |  |  |  |  |
| --- | --- | --- | --- | --- |
| **P**atient / population / problem |  |  | **P**opulation |  |
| **I**ntervention |  |  | **E**xposure |  |
| **C**omparison / control |  |  | **O**utcome |  |
| **O**utcome |  |  |  |  |
| **S**tudietyp |  |  |  |  |
|  |  |  |  |  |

# referenser till nyckelartiklar – som motsvarar sådana artiklar som du vill att sökningen ska inkludera (3-5 artiklar)

# önskat format för leverans av resultat (Endnote, pdf, word)

Endnote

# sökscheman

**2023-01-02**

**Pubmed** #1

("Gastric Bypass"[Mesh]) OR "Bariatric Surgery"[Mesh]
=32299

#2

bariatric surger*[Title/Abstract] OR gastric bypass*[Title/Abstract] OR gastric sleeve*[Title/Abstract] OR sleeve gastrectom*[Title/Abstract] OR laparoscopic gastrectom*[Title/Abstract] OR LSG[Title/Abstract] OR restrictive surger*[Title/Abstract] OR malabsorptive surger*[Title/Abstract] OR BPD[Title/Abstract] OR SG[Title/Abstract] OR biliopancreatic diversion*[Title/Abstract]
=57296

#3
#1 OR #2
=67508

#4
("Pregnancy"[Mesh]) OR "Pregnant Women"[Mesh]
=989111

#5
pregnanc*[Title/Abstract] OR pregnant[Title/Abstract]
=589303

#6
#4 OR #5
=1112893

#7
#3 AND #6
=2718

#8
((("Intensive Care, Neonatal"[Mesh]) OR "Gestational Age"[Mesh]) OR "Hypoxia-Ischemia, Brain"[Mesh]) OR "Congenital Abnormalities"[Mesh]
=736660

#9
time after operation[Title/Abstract] OR "weight loss" after operation[Title/Abstract] OR perinatal outcome*[Title/Abstract] OR perinatal complication*[Title/Abstract] OR perinatal morbidit*,[Title/Abstract] OR gestational age[Title/Abstract] OR gestational ages[Title/Abstract] OR birthweight[Title/Abstract] OR birth weight[Title/Abstract] OR congenital anomal*[Title/Abstract] OR SGA[Title/Abstract] OR LGA[Title/Abstract] OR hypoxic ischemic encephalopath*[Title/Abstract] OR HIE[Title/Abstract] OR seizures neonatal intensive care unit[Title/Abstract] OR NICU[Title/Abstract] OR GWG[Title/Abstract] OR gestational weight gain[Title/Abstract] OR WMD[Title/Abstract] OR weighted mean difference[Title/Abstract]
=206427

#10
#8 OR #9
=883153

#11
#7 AND #10
=1402

#12
("2021/04/26"[Date - Entry] : "3000"[Date - Entry])
=2588145

#13
#11 AND #12
=142

#14
"case report"[Title/Abstract]
=386084

#15
#13 NOT #15
=**141 references**

**Embase (Elsevier)**
#1
'gastric bypass surgery'/exp OR 'gastric sleeve'/exp OR 'bariatric surgery'/exp OR 'roux-en-y gastric bypass'/exp OR 'bariatric surger*':ab,ti OR 'gastric bypass*':ab,ti OR 'gastric sleeve*':ab,ti OR 'sleeve gastrectom*':ab,ti OR 'laparoscopic gastrectom*':ab,ti OR lsg:ab,ti OR 'restrictive surger*':ab,ti OR 'malabsorptive surger*':ab,ti OR bpd:ab,ti OR sg:ab,ti OR 'biliopancreatic diversion':ab,ti
=105726

#2
'pregnancy'/exp OR 'pregnant woman'/exp OR pregnanc*:ab,ti OR pregnant:ab,ti
=1138762

#3
#1 AND #2
=4110

#4
'newborn intensive care'/exp OR 'congenital disorder'/exp OR 'hypoxic ischemic encephalopathy'/exp OR 'gestational age'/exp OR 'time after operation':ab,ti OR 'weight loss after operation':ab,ti OR 'perinatal outcome*':ab,ti OR 'perinatal complication*':ab,ti OR 'perinatal morbidit*':ab,ti OR 'gestational age':ab,ti OR 'gestational ages':ab,ti OR birthweight:ab,ti OR 'birth weight':ab,ti OR 'congenital anomal*':ab,ti OR sga:ab,ti OR lga:ab,ti OR 'hypoxic ischemic encephalopath*':ab,ti OR hie:ab,ti OR 'seizures neonatal intensive care unit':ab,ti OR nicu:ab,ti OR gwg:ab,ti OR 'gestational weight gain':ab,ti OR wmd:ab,ti OR 'weighted mean difference':ab,ti
=1981371

#5
#3 AND #4
=2351

#6
#5 AND [26-04-2021]/sd NOT [03-01-2023]/sd
=379

#7
'case report':ab,ti
=524837

#8
#6 NOT #7
=**370 references

Scopus (Elsevier)**#1
TITLE ( "gastric bypass*" OR "gastric sleev*" OR "bariatric surger*" OR "sleeve gastrectom*" OR lsg OR "restrictive surger*" OR "malabsorptive surger" OR "biliopancreatic diversion*" OR lsg OR bpd OR sg OR gs ) AND PUBYEAR > 2020
=18135

#2
TITLE ( pregnanc* OR pregnant ) AND PUBYEAR > 2020 AND PUBYEAR > 2020
=28223

#3
#1 AND #2
=**118 references**

Due to problems when combining the searches in Scopus I only add searches on title. The numbers when adding abstracts/keywords are too high and cannot be correct. The time limit set by the database is 2020-.

**CINAHL (EBSCO)**

#1
MM bariatric surgery OR MM gastric bypass OR TI ( bariatric surger* OR gastric bypass* OR gastric sleev* OR GS OR sleeve gastrectom* OR LSG OR restrictive surgery OR malabsorptive surgery OR BDP OR SG OR biliopancreatic diversion* ) OR AB ( bariatric surger* OR gastric bypass* OR gastric sleev* OR GS OR sleeve gastrectom* OR LSG OR restrictive surgery OR malabsorptive surgery OR BDP OR SG OR biliopancreatic diversion* )
=15764

#2
MH (MH "Pregnancy+") OR TI ( pregnanc* OR pregnant ) OR AB ( pregnanc* OR pregnant )
=294421

#3
#1 AND #2
=550

#4
( (MM "Neonatal Intensive Care Nursing") OR (MM "Intensive Care Units, Neonatal") OR (MM "Hypoxia-Ischemia, Brain, Neonatal") OR (MM "Gestational Age") ) OR TI ( time after operation OR weight loss after operation OR perinatal outcome* OR perinatal complication* OR perinatal morbidit* OR gestational age OR gestational ages OR birthweight OR "birth weight" OR congenital anomal* OR congenital abnormalt* OR SGA OR LGA, OR hypoxic ischemic encephalopath* OR hypoxic-ischemic encephalopath* OR HIE OR seizures and neonatal intensive care unit OR NICU ORGWG OR gestational weight gain OR WMD OR weighted mean difference ) OR AB ( time after operation OR weight loss after operation OR perinatal outcome* OR perinatal complication* OR perinatal morbidit* OR gestational age OR gestational ages OR birthweight OR "birth weight" OR congenital anomal* OR congenital abnormalt* OR SGA OR LGA, OR hypoxic ischemic encephalopath* OR hypoxic-ischemic encephalopath* OR HIE OR seizures and neonatal intensive care unit OR NICU ORGWG OR gestational weight gain OR WMD OR weighted mean difference
=93771

#5
#3 AND #4
=150

#6
#3 AND #4
Limiters - Published Date: 20210101-20221231
=**23 references**

**Total number of references**

**Before deduplication: 652**

**After deduplication: 384**
