# Supplementary material for: Adverse perinatal outcomes after Roux-en-Y Gastric Bypass vs. Sleeve Gastrectomy: a systematic review
Source: BMC Pregnancy Childbirth. 2023 Aug 2;23:557. doi: 10.1186/s12884-023-05515-7 (PMC10394842; doi:10.1186/s12884-023-05515-7)
Supplement: Supplementary file 3 — Additional file 3: Table S3. References of reports with no accessible full text [file 12884_2023_5515_MOESM3_ESM.docx]

**Table S3.**

**References of reports with no accessible full text**

1. Abdallah, A. Impact of bariatric surgery ¨modified sleeve gastrectomy with loop bipartition¨ (SH-LB) on obstetric. International Journal of Gynecology and Obstetrics 2018;143():250-251.
2. Holloman, C. Carlan, S. No improved pregnancy outcomes noted in women with gastric bypass compared to those with class III obesity. Obstetrics and Gynecology 2016;127():37-38.
3. Mottin, C. Bebber, FE. Padoin, A. Rodrigues, MT. Rizzolli, J. Casagrande, DS. Klaesener, RE. Moretto, M. Repetto, G. Pregnancy after bariatric surgery: 30 pregnancies follow-up in a multidisciplinary team. Obesity Surgery 2009;19(8):989-990.
4. Rebollar, MF. Mosti, M. Chapa, ME. Sanchez, H. Gonzalez, L. Cordero, SL. Mier Y Terán, N. Herrera, M. Obstetrical and perinatal outcomes of pregnancy following Roux-en-Y gastric bypass. Surgery for Obesity and Related Diseases 2011;7(3):359-360.
